# Feature Analysis of Smart Shoe Sensors for Classification of Gait Patterns

**DOI:** 10.3390/s20216253

**Published:** 2020-11-02

**Authors:** Unang Sunarya, Yuli Sun Hariyani, Taeheum Cho, Jongryun Roh, Joonho Hyeong, Illsoo Sohn, Sayup Kim, Cheolsoo Park

**Affiliations:** 1 Department of Computer Engineering, Kwangwoon University, Seoul 01897, Korea; unangsunarya@telkomuniversity.ac.id (U.S.); yulisun@tass.telkomuniversity.ac.id (Y.S.H.); 2 School of Applied Science, Telkom University, Bandung 40257, Indonesia; 3 Department of Intelligent Information and Embedded Software Engineering, Kwangwoon University, Seoul 01897, Korea; taeheumcho89@kw.ac.kr; 4 Human Convergence Technology R&D Department, Korea Institute of Industrial Technology, Ansan 15588, Korea; ssaccn@kitech.re.kr (J.R.); freegore@kitech.re.kr (J.H.); 5 Department of Computer Science and Engineering Seoul National University of Science and Technology, Seoul 01811, Korea; isohn@seoultech.ac.kr

**Keywords:** smart shoes, gait analysis, feature analysis, pronation, supination, accelerometer, gyroscope, pressure sensor

## Abstract

Gait analysis is commonly used to detect foot disorders and abnormalities such as supination, pronation, unstable left foot and unstable right foot. Early detection of these abnormalities could help us to correct the walking posture and avoid getting injuries. This paper presents extensive feature analyses on smart shoes sensor data, including pressure sensors, accelerometer and gyroscope signals, to obtain the optimum combination of the sensors for gait classification, which is crucial to implement a power-efficient mobile smart shoes system. In addition, we investigated the optimal length of data segmentation based on the gait cycle parameters, reduction of the feature dimensions and feature selection for the classification of the gait patterns. Benchmark tests among several machine learning algorithms were conducted using random forest, k-nearest neighbor (KNN), logistic regression and support vector machine (SVM) algorithms for the classification task. Our experiments demonstrated the combination of accelerometer and gyroscope sensor features with SVM achieved the best performance with 89.36% accuracy, 89.76% precision and 88.44% recall. This research suggests a new state-of-the-art gait classification approach, specifically on detecting human gait abnormalities.

## 1. Introduction

People over the age of 45 years experience foot pain regularly, and about two-third of these are at least mild impairment in any aspect of their foot conditions related to activities in their daily lives [1]. In such cases, foot biomechanics play an important role in the development and progression of foot pain during daily tasks, including walking. Moreover, Menz et al. [2] stated that a pronated foot significantly increased the probability of the development of generalized foot and heel pains. Foot supination occurs when body weight falls on the outer edges of the feet, while overpronation occurs when the foot falls more inward or downward [3]. Those who have the supination and overpronation too frequently are at high risk of developing foot disorders and symptoms including ankle and feet pain, plantar fasciitis, foot fatigue, etc. [2]. Additionally, these conditions might worsen during walking, running or standing for a long time.

Automatic detecting approaches of these abnormalities in early stage could correct the walk posture and avoid further injuries, one of which is gait analysis. Gait analysis is conducted as an efficient clinical method for a wide variety of applications such as neurological diseases assessment [4,5,6], prevention of falling accidents [7,8,9], orthopedic disorder diagnosis [10,11,12] and enhancement in recovery process after knee or leg related surgery on post-surgical patients [13,14]. The gait analysis is typically conducted in a specific clinical laboratory using pressure mats or vision-based equipment. This setting of the gait experiment in such a clinical laboratory has two disadvantages. Firstly, building the specific clinical laboratory with all the equipment is costly. Secondly, walking on the pressure mat hinders participants from walking naturally, and thus the experiments cannot record the real gait patterns [15]. Hence, this type of experiment is not suitable for the gait analysis which is supposed to be conducted in sufficient and comfortable environment in order for participants to walk naturally. For its accurate analysis, the experiment data are recorded during a long walking most critically under unregulated conditions.

In recent years, several works have been published to develop wearable systems in order to assess the gait patterns using different types of sensors or combination of them, such as electromyography sensors [16,17,18], gyroscopes [19,20], accelerometers [21,22,23] and pressure sensors [24,25,26]. Moreover, other gait instruments have also been used for several different purposes; for example, inertial measurement units are placed at shank and waist to classify elderly, post-stroke and Huntington’s disease using a combination of support vector machine (SVM) and hidden Markov model (HMM) [27]. Similarly, Gao et al. used the inertial sensor placed in waist and ankle to differentiate normal and abnormal gaits by employing a deep neural network [28]. However, the placement of the sensors in the waist and ankle would not be convenient to keep wearing during a daily life. The combination of accelerometers, gyroscopes and pressure sensors equipped with insoles were utilized to classify several types of gait patterns such as walk, run, stair climb up and stair climb down [29]. While this study showed high accuracy with over 90% classification, the abnormality of a gait was not addressed.

Unlike the previous studies that focused on normal gait patterns such as walking, running, climbing up stairs and climbing down stairs, this study used data collected from participants wearing smart shoes to detect normal and four abnormalities, namely pronation, supination, unstable left foot and unstable right foot. Smart shoes were equipped with an accelerometer, a gyroscope and four pressure sensors, which were placed on each outsole to encourage people to wear shoes comfortably and walk naturally. Extensive feature analyses were then conducted to find the best sensor combination and the optimal number of significant features to be used for the gait classification. These analyses included the investigation of different sensor combinations and different data segmentations based on the gait cycle parameters. In addition, significant features were selected based on their information gains, and the feature number was reduced using principal component analysis (PCA) [30]. These final features were utilized for the gait classification task with the random forest (RF), k-nearest neighbor (KNN), logistic regression and SVM algorithms. The objective of this study was to find the best combination of the sensors producing significant features by conducting feature analysis and performing five-class classification.

**Figure 1:**
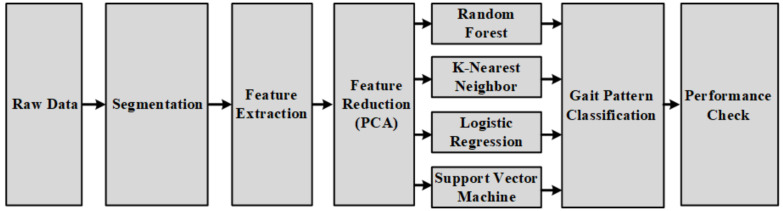
Block diagram for the identification of the gait patterns using pressure, three-axis accelerometer and three-axis gyroscope sensors. Four machine learning algorithms were applied—random forest, k-nearest neighbor, logistic regression and support vector machine—to classify five gait types.

Figure 1 shows the overall experiment process of the gait classification. Raw data from the smart shoes sensors were segmented, and nine statistical feature extracting method were applied. To avoid the complexity of the model due to high dimension of multiple feature spaces, principal component analysis (PCA) was initialized for a feature reduction. Four machine learning algorithms were applied to classify gait patterns: random forest, k-nearest neighbor, logistic regression and support vector machine. In the end, their performances were evaluated in terms of accuracy, precision and recall, as explained in Section 2.8.

## 2. Materials and Methods

### 2.1. Data Acquisition

**Figure 2:**
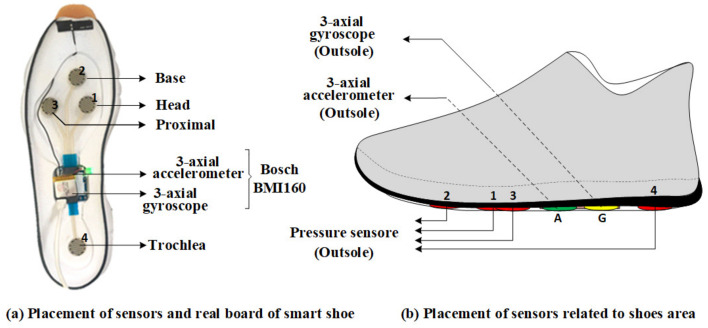
The placement of sensors: eight pressure sensors were mounted on the outsole of the shoes as well as two Bosch BMI160 sensors. Each Bosch BMI160 sensor consists of a three-axis accelerometer and a three-axis gyroscope, respectively. There were 12 sensors in total mounted no the outsole of both the left and the right shoes. (**a**) The position of sensors on the real board of smart shoe. (**b**) The position of sensors outsole of the smart shoes.

There were 18 healthy participants (18 men, age: 26.2 ±3.9 years; height: 176.2 ±5 cm; body mass: 73.1 ±8.8 kg) recruited for this study, and their average shoe size was 267.5 ±7.9 mm. Each participant wore shoes equipped with three types of sensors that were mounted in a particular position of the shoe, as can be seen in Figure 2. Four pressure sensors, a three-axis accelerometer and a three-axis gyroscope sensor were mounted on each shoe outsole. The pressure sensor produces one-channel data, while the accelerometer and gyroscope sensors each yield three-channel data. Therefore, a 20-channel dataset was acquired for one pair of shoes.

Each participant in the smart shoes was instructed to walk on the treadmill and mimic five gait types: normal, unstable left, unstable right, supination and pronation. The normal gait was conducted for 3 min while the four other gait for 1 min each.

The normal gait without any musculoskeletal disorder could be defined when participants have a natural symmetry of body between left and right sides, and they are often endured to the left or right sides in the situation of left and right instability [31]. The term supination (toe-out-gait) is often used for the movement of the upper limb. When a participant’s moves cause more body mass to be endured by the lateral border due to the body mass and the time gap between heel and metatarsal contacts to the ground decreases, it is called supination. On the other hand, pronation (toe-in-gait) is the opposite movement to supination [32].

We trained participants who have normal gait (without musculoskeletal disorders) to perform the abnormal gaits. The definition and characteristic of each abnormal gait were delivered to all participants to ensure the participants followed all the instructions: In the case of unstable left and right, compared to normal walking, the step length of the target foot is shortened and the ratio of stance/swing is changed from normal walking. In supination (Toe-out-gait), the time gap between heel and metatarsal contacts to the ground is reduced compared to that of the normal walking where the metatarsal touches the ground after the heel-contact owing to the valgus of the toes of both feet. In the case of pronation (Toe-in-gait), the toes of both feet are varus inward, and thus the weight is shifted to the outside of the foot.

### 2.2. Segmentation

**Figure 3:**
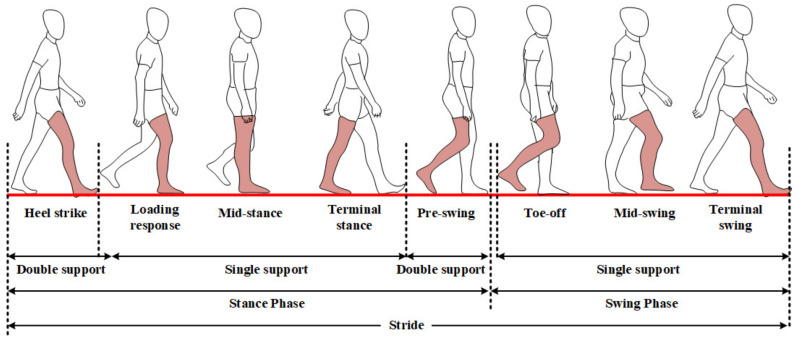
A stride consisting of multiple actions of legs and feet.

Data segmentation is a crucial process for the classification of the gait patterns into normal and abnormal walks, which was conducted based on the length of stride, step, stance phase, swing phase, left single limb support, right single limb support and double limb support. Figure 3 illustrates a stride consisting of multiple actions of legs and feet. The length of one stride is defined as the period from one heel-strike on the right foot to the next one. The length of one step could be estimated as half of a stride period. The length of the stance and swing phases are estimated by the duration from the heel-strike on the right foot to the pre-swing on the right foot, as well as from toe-off on the right foot to the hill-strike on the right foot [33].

### 2.3. Feature Extraction

**Figure 4:**
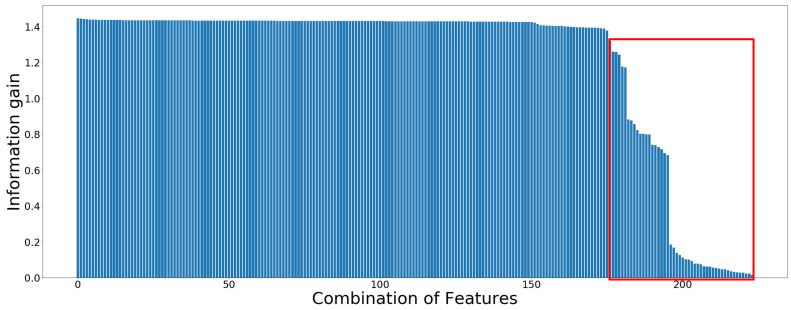
Information gain calculated from 224 features of 20 channels sensor data.

As described in Table 1, the correlation, mean, standard deviation, kurtosis, crest factor, skewness, entropy and spectral flux features were extracted from all six sensor data. To find the optimal feature sets for the gait pattern analysis, 172 features combinations of the 20-channel sensor data were considered (see Figure 4). These features were chosen based on analysis obtained from the information gain.

### 2.4. Feature Selection

Feature for the gait pattern classification are extracted from 20-channel sensor data (10 channels each on the right shoe and the left shoe), yielding 172 high dimension feature space, which could cause long processing time or even overfitting [35,36]. Among all these features, some features could be significant for the performance of the gait pattern classification, and the others might not. To overcome this issues, the information gain of each feature was calculated to investigate its significance for the classification performance [37]. Based on the information gain, only the meaningful features were utilized to classify the gait patterns.

### 2.5. Feature Reduction

Principal component analysis (PCA) was applied to reduce the high dimension of feature space. Unlike feature selection, feature reduction by PCA projects the features onto multiple orthogonal domains. By selecting the subset of principle components that have high variance, the feature space dimension will be reduced. PCA was applied to the standardized features, that is, zero-mean and unit variance, in order to prevent some features from dominating the others owing to their large scales [38].

### 2.6. Classifier

#### 2.6.1. Random Forest

Random forest (RF) is one of the most popular machine learning algorithms for regression and classification tasks [39,40]. It is composed of multiple decision trees and considered as an ensemble of decision trees, that is, combining weak learners to build a more robust model. Random forest algorithm uses bagging method to prevent overfitting problem, resulting in generalization of the model [41,42].

#### 2.6.2. K-Nearest Neighbor

K-nearest neighbor (KNN) is one of the supervised learning algorithms used for classification problems and is based on non-parametric method. KNN algorithm works with the steps as follows: choose the number of *k* (class number) and a distance metric; find the nearest neighbor for each sample; and assign class labels based on a majority vote [41].

#### 2.6.3. Logistic Regression

Logistic regression is the most widely used algorithm for classification problems. It is a linear model for binary classification that can be extended to multiple class classification. This algorithm calculates the probabilities of class labels using a logistic function as follows [41]:

(1) $$\phi \left(x\right)=\frac{1}{1+e^{-f(x)}}$$

where *x* is an input and f(x) is a linear function corresponding to the input *x*.

#### 2.6.4. Support Vector Machine

A support vector machine (SVM) is also one of the most popular machine learning algorithms for classification problems, which tries to find a hyperplane to maximize the distance between different classes with adjusting margin, defined as the distance between the decision boundary and the closest training samples (support vectors) [41]. For a nonlinear process of SVM to improve the classification performance, the kernel trick is applied [43]. The radial basis function (RBF) kernel function described in Equations (2) and (3) was used for gait pattern analysis.

(2) $$k\left(x^{\left(i\right)},x^{\left(j\right)}\right)=exp\left(-\gamma {∥x^{\left(i\right)},x^{\left(j\right)})∥}^{2}\right)$$

(3) $$\gamma =\frac{1}{2\sigma^{2}}$$

where ${∥x^{\left(i\right)},x^{\left(j\right)})∥}^{2}$ is the squared Euclidean distance between two data points $x^{\left(i\right)}$ and $x^{\left(j\right)}$.

### 2.7. Experiment Setting

The dataset from the 18 participants was separated into training and testing sets. The performance was evaluated using leave-one-out cross-validation, where one participant’s data were used for the testing set and the rest for the training. Random forest, k- nearest neighbors, logistic regression and SVM algorithms were applied to classify the input data. We set the SVM (C = 1, degree = 3, gamma = scale, and kernel = rbf), RF (100 number of estimators, gini criterion, 15 minimum samples split, 30 maximum depth and 1 minimum sample leaf) and KNN (k = 5) parameters.

### 2.8. Evaluation Metrics

The performance of the algorithm was evaluated using three performance metrics: accuracy, precision and recall. The accuracy was calculated as follows:

(4) $$Accuracy=\frac{TP+TN}{TP+TN+FN+FP}$$

where TP refers to the number of predictions that the classifier correctly predicts one out of five gait classes, FP is the total number of prediction when the classifier incorrectly predicts gait classes, FN is the total number of incorrect prediction for a certain true gait class and TN is the total score excluding TP, FP and FN. The precision score is the true positive (TP) rate with respect to the total number of prediction score related to all actual classes [34,44]. It is calculated using the equation below:

(5) $$Precision=\frac{TP}{TP+FP}.$$

The recall value is the rate of TP among all the certain true class, which is formulated as [34,44].

(6) $$Recall=\frac{TP}{TP+FN}.$$

This paper addresses a multi-class classification problem; thus, the average, precision and recall are calculated using weighted average by computing the metric independently for each class and then taking the average.

## 3. Results

The performance metrics of accuracy, precision and recall were produced depending on the feature combinations of three sensor data, which were yielded using random forest, k-nearest neighbor, logistic regression and SVM.

### 3.1. Feature Selection and Reduction

All nine statistical features, described in Table 1, including zero crossing and maximum value were extracted from raw data. There are 224 feature produced from the 20 sensor data, which are represented in the x-axis of Figure 4. Figure 4 displays the information of all 224 feature with respect to the gait pattern labels. The higher the information gain is, the more information for the classification task could be expected [37]. The last 52 features, including power feature of accelerometer and gyroscope, all max and zero-crossing features, highlighted with a red rectangle, have relatively lower level of information gains compared with the rest of them, and thus they were discarded for the analysis. The feature with the highest information gain was the correlation between channel *X* and *Y* of gyroscope on the right foot.

Table 2 elaborates the number of statistical features from the 20-channel sensor data. The – sign indicates the ignored features from the sensor data.

The correlation feature was calculated for two-channel data. In the case of the pressure sensor, the correlation of the pressure sensor data were calculated between two-channel data in each shoe, and thus correlation features were yielded from four pressure sensors in each shoe, resulting in 12 for both shoes. A three-axis accelerometer as well as the gyroscope sensor generates three correlation features. The rest of the features have the same number of channels.

**Figure 5:**
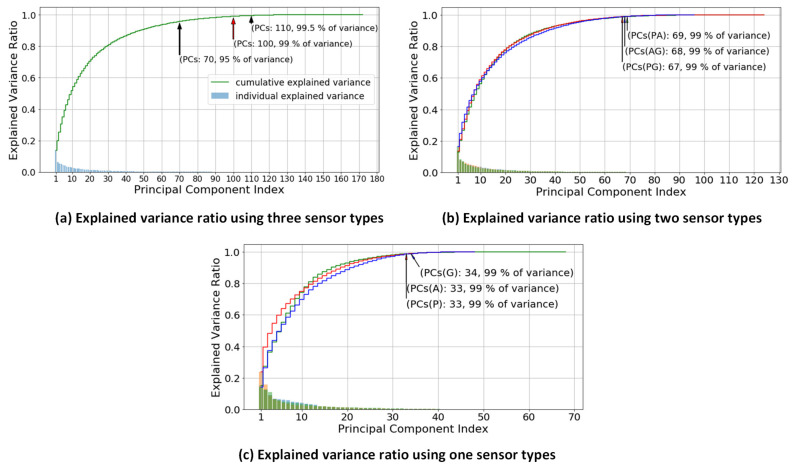
Individual and cumulative explained variances for determining the optimal number of principal components. The cumulative explained variance shows the accumulation of variance for each principal component number. The individual explained variance describes the variance of each principal component.

These significant 172 features decided by the information gain are still a large number to demand a complex model with high computational power, and the complex model could cause overfitting problem [35,36]. Therefore, a process of feature reduction was conducted using PCA; the number of PCs was decided based on the percentage decrease in original variance, as shown in Figure 5. The PCA was performed for feature reduction by transforming the correlated gait features into a smaller number of uncorrelated gait features, also known as principal components (PCs) [45]. In the study, the transformation was explained using the cumulative explained variance that mapped the value of variance-ratio to its number of PCs. The cumulative explained variance denotes the accumulation of variance ratio corresponding to the first number of PCs, while the individual explained variance shows the variance value for each PC. A cumulative explained variance threshold of 99% is used in this study in order to obtain the optimal number of PCs.

Figure 5a describes the explained variance ratio with respect to number of PCs using three different types of sensors, and thus the first 100 PCs are the optimal number of features. Figure 5b shows the explained variance ratio with respect to number of PCs using two different types of sensors. ‘PA’ denotes Pressure Sensors and Accelerometer, ‘AG’ denotes Accelerometer and Gyroscope and ‘PG’ denotes Pressure Sensors and Gyroscope. The optimal number of PCs is 69 for PA, 68 for PG and 67 for GA. Lastly, for individual sensor types P (Pressure Sensors), A (Accelerometer) and G (Gyroscope), the explained variance ratio with respect to number of PCs is shown in Figure 5c. With the same threshold value, the optimal number of PCs of P is 34, while for both A and G it is 33.

### 3.2. Significance of Smart Shoes Sensors to Gait Pattern Analysis

**Figure A1:**
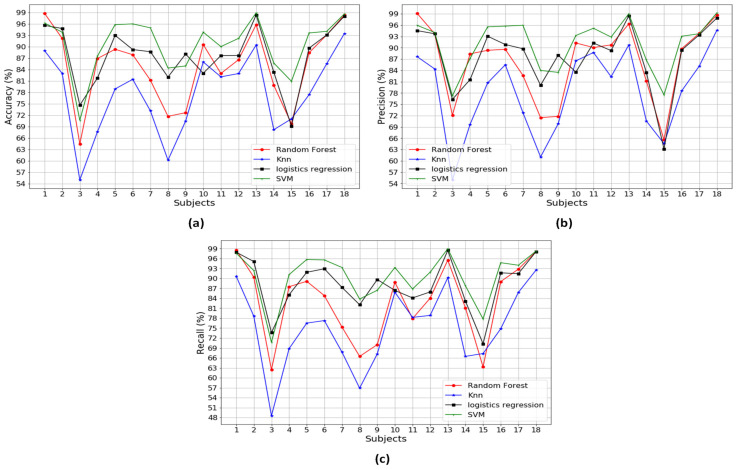
The classification performances of methods using a combination of three different types of sensors: pressure sensors, accelerometer and gyroscope: (**a**) accuracy performance; (**b**) precision performance; and (**c**) recall performance.

**Figure A2:**
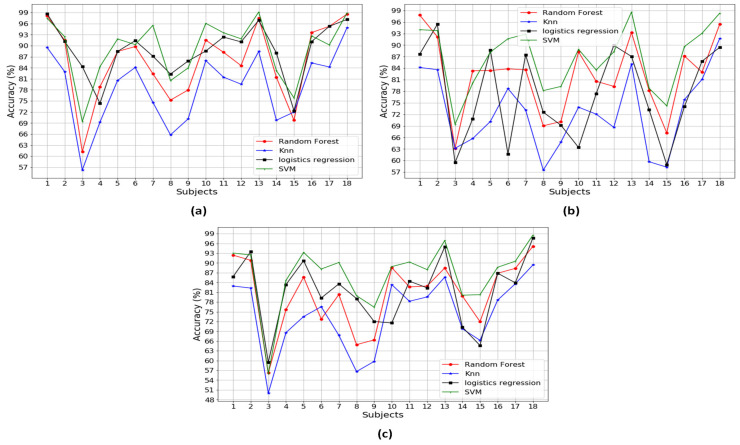
The average accuracy performance over a combination of two types of sensors: (**a**) accuracy over Pre+Acc; (**b**) accuracy over Pre+Gyro; and (**c**) accuracy over Acc+Gyro.

**Figure A3:**
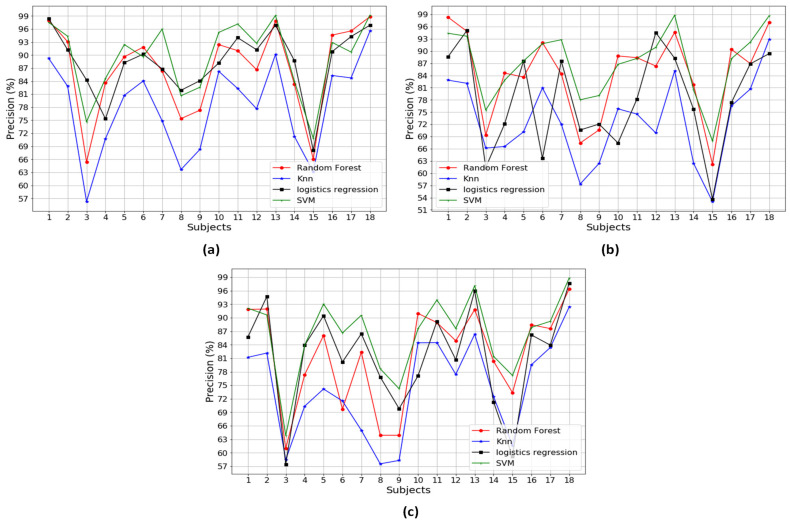
The average precision performance over a combination of two types of sensors: (**a**) precision over Pre+Acc; (**b**) precision over Pre+Gyro; and (**c**) precision over Acc+Gyro.

**Figure A4:**
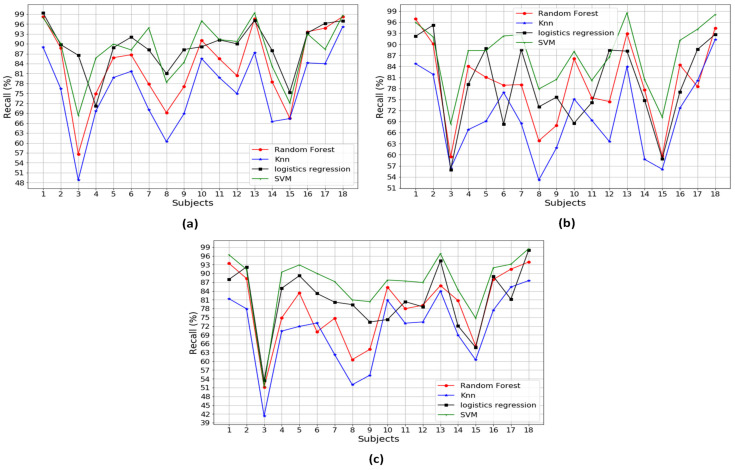
The average recall performance over a combination of two types of sensors: (**a**) recall over Pre+Acc; (**b**) recall over Pre+Gyro; and (**c**) recall over Acc+Gyro.

**Figure A5:**
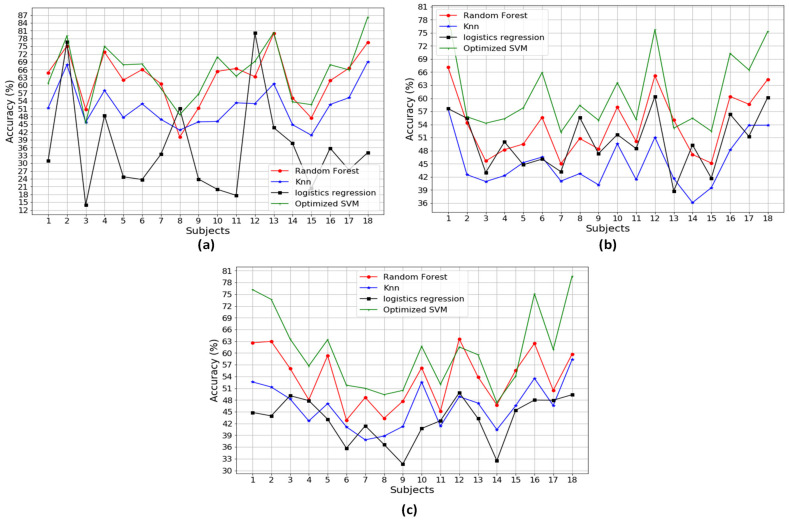
The average accuracy performance over individual types of sensors: (**a**) accuracy over pressure sensor; (**b**) accuracy over accelerometer; and (**c**) accuracy over gyroscope.

**Figure A6:**
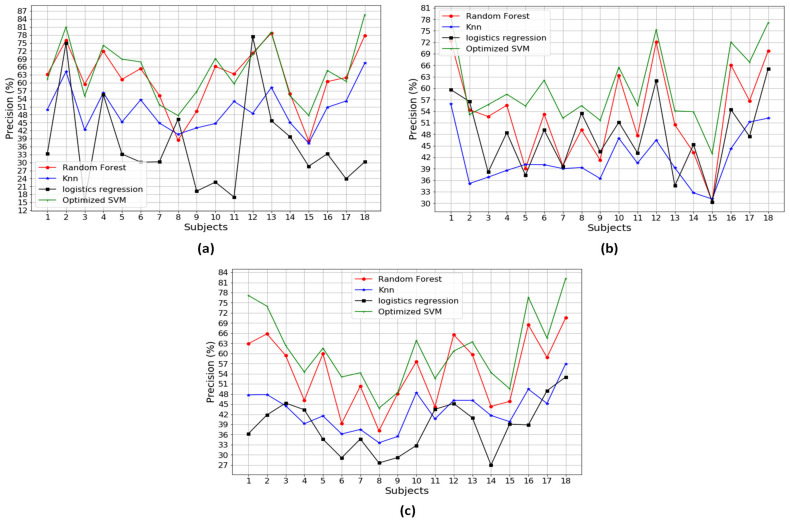
The average of precision performance over individual types of sensors: (**a**) precision over pressure sensor; (**b**) precision over accelerometer; and (**c**) precision over gyroscope.

**Figure A7:**
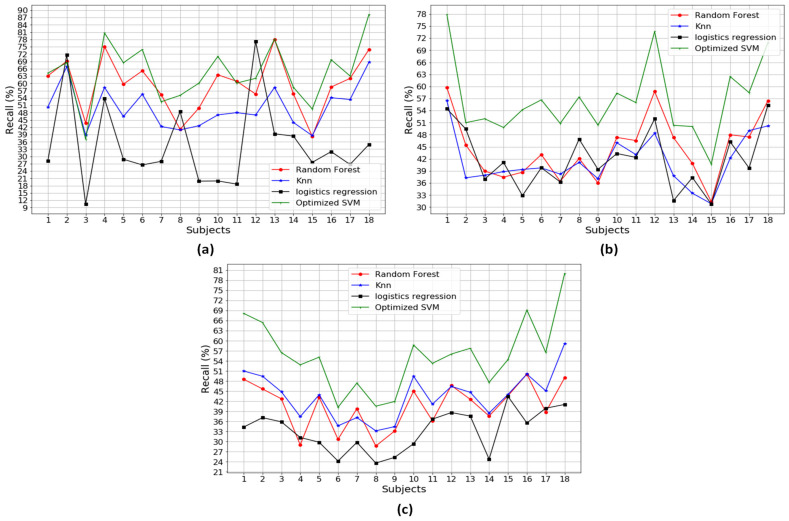
The average of recall performance over individual types of sensors: (**a**) recall over pressure sensor; (**b**) recall over accelerometer; and (**c**) recall over gyroscope.

In this part, the significance of the smart shoes sensors to the gait pattern analysis is looked into using the combination of the sensor features. Table 3 shows the averaged accuracy of the gait pattern classification using the various combinations of the sensor features across all 18 participants. Each performance of the individual participant can be found in Figure A1, Figure A2, Figure A3, Figure A4, Figure A5, Figure A6 and Figure A7. As can be seen in Figure 5, all the combinations including individual, two and three sensor features retain 99% of variance to determine the significant PCs, indicating meaningful information of all combinations of the features. The classification accuracy of five different conditions—normal, left, right, supination and pronation—was calculated using RF, KNN, logistic regression and SVM.

The SVM classifier yields higher accuracy compared to the other algorithms for all combinations of the sensor features. Even though the usages of all three sensor features produced the best accuracy, 90.64%, “Acc+Gyro” obtained comparable performance, 89.36%, which is 1.35% difference. In particular, “Acc+Gyro” requires many fewer PCs (68) than those of Pre+Acc+Gyro (100), resulting in an efficient classifier in terms of computational complexity and power. The same results could be confirmed in terms of precision in Table 4 and recall in Table 5.

Table 4 describes the performance in terms of precision, calculating true positive (TP) rate among all the predictions [34], where the SVM classifier also performs best. A combination of accelerometer and gyroscope reached the precision score of 89.67%. This precision score has 1.40% different to the precision score of Pre+Acc+Gyro, but has fewer PCs (68). It means Acc+Gyro could have a faster processing time than Pre+Acc+Gyro. Table 5 shows the recall performance; the overall performance is the best when SVM is used.

The significant difference in Table 6 is provided to show that the SVM is superior (*p* < 0.05), compared with the other algorithms in our experiments except the logistic regression. The main reason the logistic regression classifier has no significant difference with SVM is because it separates the classes using a simple linear decision boundary while the SVM utilizes a nonlinear boundary with a nonlinear kernel function of RBF [46].

In addition, it was confirmed that the multiple sensor features “Acc+Gyro” significantly improve the classification performance, compared with the single sensor features, as shown in Table 7, which was tested using the one-tailed paired sample t-test.

### 3.3. Optimal Number of Principal Components for the Classification Performances

Table 8 shows the comparison of classification performance between a combination of two and three different types of sensors based on number of PCs using the SVM method. The combinations of two and three different types of sensors have 96 and 172 PCs, respectively. We choose the optimal number of PCs from each combination by retaining 99% of its variance. The combination of two types of sensors reached the accuracy performance of 89.36%, precision of 89.76% and recall of 88.44% on the optimal number of PCs (68). The combination of three different types of sensors gained the optimal number of PCs (100) producing accuracy performance of 90.64%, with precision and recall of 91.08% and 90.55%, respectively. Even though the combination of three different types of sensors achieves a higher performance on its optimal number of PCs, on the same number of PCs, the combination of two different types of sensors reaches higher performance score than a combination of three different types of sensors.

### 3.4. Performance Gait pattern Classification on Each Segmentation Type

**Figure 6:**
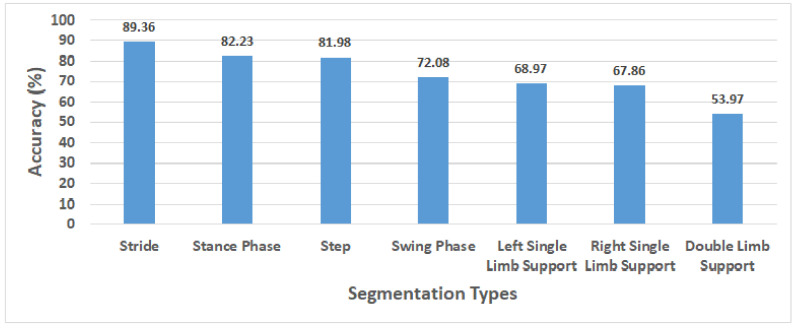
The comparison of average accuracy on Acc+Gyro sensor combination using seven different segmentation types across 18 participants. The average accuracy was measured using SVM at optimal PCs number (68) on PCA.

In this study, we also confirmed the classification performance based on six other segmentation types as a comparison to the stride segmentation using Acc+Gyro as a proposed sensor combination, as shown in Figure 6. In this figure, the sequence of segmentation types starting from stride to double limb support shows a trend of decreasing average accuracy along with the shorter length of the segmentation data. The SVM as the best algorithm during the implementation was used to calculate the average accuracy on each segmentation type across 18 participants. Based on our experiment, the length of segmentation type has an effect to the performance results. The stride segmentation, which has longer period than the step segmentation, shows higher performance (89.36%) than the step segmentation (81.98%), while the double limb support as the shortest one obtained the lowest performance (53.97%). In Figure 3, it can be seen that the period of the stride segmentation is twice as long as that of the step segmentation, helping the sensors collect more information during walking, which could be a reason the longer segmentation type obtained higher performance than the lower segmentation types.

## 4. Discussion

In this section, both feature analysis and sensor significance are further discussed. The feature analysis involving feature selection and reduction have an important role in reducing processing time and preventing the model’s complexity for gait pattern classification. Firstly, the information gain is used to select 172 prominent features out of 224 features. However, the selected number of features is too large to be computed in the classification algorithm, so that PCA is applied for the reduction of the features dimensions. PCA provides low dimensional approximations to the data with projecting the data orthogonally onto linear subspaces.

Figure 5 shows the variance ratio with respect to its principal components of each sensor combination. The variance of each PC represents the contribution rate with respect to the performance of algorithms [38]. The curve of cumulative explained variance illustrates the variance ratio for its number of PCs. Besides that, each point of the variance ratio in the curve would affect the high or low performance results of the algorithms. The lower is the chosen variance ratio, the lower is the number of PCs utilized, and consequently the performance also deteriorates. This is trade-off between the number of PCs and its performance algorithms. The lower number of PCs demands lower computation time, and thus it is necessary to find the optimal number of PCs. In Figure 5, we retain 99% of the variance from the original dimension space as the optimal number of PCs. Additionally, Table 8 describes the effect of different number of selected PCs on the classification performance. In the case of “Acc+Gyro’, the optimal number of PCs (68) gives 89.36% accuracy which is comparable to the accuracy using the maximum number of PCs (96) with 89.30%. This number of PCs, 68, is only 30% of the original, 224. Similarly, in the case of “Acc+Gyro+Press’, the optimal number of PCs (100) gives the accuracy as high as that using maximum number of PCs (172). These two case results demonstrate that the selected optimal PCs maintain the classification performance with a lower feature dimension than the original.

The sensor significance analysis was conducted to find the optimal combination of sensors for the development of low-cost smart shoes. Table 3 describes the contribution of each sensor and the combination of sensors to the classification performance. It clearly demonstrates that the smart shoes with an individual type of sensor provided around 60% accuracy only. Interestingly, the combinations of two and three sensors with the SVM classifier yielded a comparable accuracy from 86% to 90%. The combination of “Acc+Gyro” gives the most comparable accuracy, 89.36%, to the combination of all the three sensors, 90.64%. Moreover, in terms of feature space dimension shown with the number of PCs, the combination of two sensors required fewer feature dimensions than those of three sensor combination. Since this study aims to develop low-cost smart shoes, indeed using the “Acc+Gyro” sensors is favorable to be chosen than those using all three sensors regarding the cost of materials and model complexity.

The selection of gait segmentation types has an important role to reach the best performance classification. In Figure 6, we sort the segmentation types based on the length of step during the participants walking from the longest to the shortest ones. The longer the step is, the more information it has. The results show the performance of gait classification using the stride reached the highest score (89.36%) followed by the stance phase (82.23%), step (81.98%), swing phase (72.08%), left single limb support (68.97%), right single limb support (67.86%) and double limb support (53.97%). The stance phase, slightly different from the step in the length of segmentation, shows the next highest performance and slightly higher performance than that of the step. Meanwhile, the swing phase, the left single limb support and the right single limb support reached performances lower than that of the step. The double limb support with the shortest segmentation length obtained the worst performance due to the least information.

Figure A1, Figure A2, Figure A3, Figure A4, Figure A5, Figure A6 and Figure A7 in the Appendix A illustrate the classification performances of the algorithms over 18 participants. Figure A1 describes the classification performances when using a combination of three different types of the sensors. It is noted that SVM outperformed the other methods, with the highest performance, 99.15% accuracy, 99.34% precision and 99.08% recall. Figure A2, Figure A3 and Figure A4 show the classification performances when using a combination of two different types of the sensors, that is a combination of Pre+Acc, Pre+Gyro, and Acc+Gyro, respectively. Based on the analysis in Table 3, the combination of Acc+Gyro using SVM is the best with the averaged accuracy of 89.36%, precision of 89.76% and recall of 88.44%. Figure A5, Figure A6 and Figure A7 describe the classification performances when using the individual sensor. Compared with the performances of the combinations using two or three types of sensors, those using the individual sensor were worse. This would be because the methods using an individual sensor obtain insufficient information and thus produce poor performances.

By analyzing the confusion matrix in Table 9, the error rate from those three participants (3, 8, and 15) are relatively high (25.4%, 19.0% and 32.3%). We compared them with the other participants who yielded higher performance (Participants 5, 13 and 18), but had relatively lower error rates (7.6%, 0.9% and 0.8%). There are two reasons for these: First, even if they are healthy participants, the walking ratio (stance/swing) could be unbalanced [47]. Most of the errors can be inferred as they are distributed in unstable left/right compared to normal gait. Second, it might not be easy for those normal participants to mimic the abnormal gait even though he was trained. We have included these interpretations about the limitations of the experiments. Based on the results, the total number of each gait type might be different due to the segmentation process. We used a threshold to decide the start and the stop of one segment. Based on the experiment, it was empirically found that 40 is the best threshold.

Table 10 shows the performance of methods used in related studies. Dominguez et al. proposed neural network (NN) model to identify gait types [48]. Even though the model obtained an accuracy of 90%, this study only classified two gait types, namely supination and pronation with total participants six people. Jiang et al. [49] identified two activities, walking and jogging, using a convolutional neural network (CNN) on eight different people and obtained 92.5% accuracy. Hayashi et al. [50] used the SVM algorithm to classified healthy–unhealthy patients. Using the same method, Begg et al. [43] conducted gait analysis and differentiated into young and old classes. Asymptomatic and osteoarthritis was differentiated from gait pattern using polynomial representation and wavelet by Mezghani et al. [51]. In the study, they utilized a 3D ground reaction force to acquire the signal from the participants. Zeng et al. [52] identified healthy and anterior cruciate ligament patients using RBF-NN and reached 93.47% of accuracy.

Zhang et al. [53] proposed the combination methods to recognize old and young people from gait patterns. Using Counter+HMM, Silhouette+HMM, Counter+Naive Bayes and Silhouette+Naive Bayes accuracy performances of 83.33%, 76.24%, 65.85%, and 63.28%, respectively, were obtained. In our study, we classified gait types into five classes with an accuracy of 89.36%. Our method is comparable to other related studies due to the higher number of classes but still showing good performance.

The practical application of this study could be implemented as wearable smart shoes in daily activities as early detection of foot abnormality. This technology has the possibility to be part of the development of the Internet of things in the future [54]; by wearing smart shoes, the gait signals from the patients could be captured for further analysis. The future study of smart shoes could be conducted to identify an abnormality or particular disease in our body, by putting on the sensors on the specific area of the foot that has a relation with the specific organs [55,56].

In this study, the participants were not experiencing any gait disorder. All participants were healthy graduate and undergraduate students without musculoskeletal disorders. All participants spent their time sitting and studying in the classroom. They did not use their own vehicle when going out, but more often went on foot. They just mimicked four abnormal gait types under the supervision of the experts. Before capturing their gait signals, the participants were trained to mimic each gait type. The gait data were captured within 3 min, hence the selection of segmentation types should be considered for further study.

## 5. Conclusions

This paper provides a thorough feature analysis using data collected from 18 participants wearing smart shoes to detect the normal gait and four gait abnormalities such as supination, pronation, unstable left foot and unstable right foot. Through the feature analysis, the optimum combination of sensors with significant features for the gait classification was obtained, which is useful in developing efficient and less computation-intensive smart shoes. The gait classification was conducted using four different algorithms: RF, KNN, logistic regression and SVM algorithms. Based on extensive experiments, the combination of the accelerometer and gyroscope with the stride segmentation using SVM achieves the best performance. The state-of-the-art method proposed herein can be used for developing efficient and low-cost smart shoes for gait classification.

## Figures and Tables

**Table 1:** The nine features in time–frequency domains.

No	Feature	Equation	Description
1	Correlation	$Corr\left(x,y\right)=\frac{1}{N-1}\sum_{i=1}^{N}\frac{\left(x_{i}-\bar{x}\right)\left(y_{i}-\bar{y}\right)}{Std(x)Std(y)}$	Relation between two sensor channel data (*x* and *y*-axis).
2	Mean	$\bar{x}=\frac{1}{N}\sum_{i=1}^{N}x\left(i\right)$	Average of data *x*(*i*) with respect to data length of an epoch (N).
3	Standard Deviation	$Std\left(x\right)=\sqrt{\frac{1}{N-1}\sum_{i=1}^{N}{\left(x\left(i\right)-\bar{x}\right)}^{2}}$	Variation of a channel data.
4	Kurtosis	$K\left(x\right)=\frac{E{\left(x-\bar{x}\right)}^{4}}{Std{\left(x\right)}^{2}}-3$	The tailedness of the probability distribution of one channel data using the 4th central moment with respect to variance [34].
5	Crest Factor	$CF\left(x\right)=\frac{max(x(i))}{\sqrt{\frac{1}{N-1}\sum_{i=1}^{N}x{\left(i\right)}^{2}}}$	How much extreme the peak of data is by measuring the ratio of the maximum value of the one sensor channel data to the effective value of the data.
6	Skewness	$\gamma \left(x\right)=E\left[{\left(\frac{x-\bar{x}}{Std(x)}\right)}^{2}\right]$	An asymmetry measure of probability distribution of one channel data to its mean.
7	Entropy	$S\left(x\right)=-\sum_{i}p_{x}\left(i\right)ln\left(p_{x}\left(i\right)\right)$	Total probability mass function of one channel data [34].
8	Spectral Flux	$SF\left(t\right)=\sum_{i=2}^{N}{\left(x_{t}\left(i\right)-x_{t-1}\left(i\right)\right)}^{2}$	The total difference between the successive data of one channel data.
9	Power	$P\left(x\right)=\frac{1}{N}\sum_{i=1}^{N}{(x(i))}^{2}$	The average energy of one channel data.

**Table 2:** The number of features for each sensor.

Feature’s Name	Pressure Sensor	Accelerometer	Gyroscope
Correlation	12	6	6
Mean	8	6	6
Standard deviation	8	6	6
Kurtosis	8	6	6
Crest factor	8	6	6
Skewness	8	6	6
Entropy	8	6	6
Spectral flux	8	6	6
Power	8	$\;6^{-}$	$\;6^{-}$
$Zerocrossing^{-}$	$\;8^{-}$	$\;6^{-}$	$\;6^{-}$
$Maxvalue^{-}$	$\;8^{-}$	$\;6^{-}$	$\;6^{-}$

${}^{-}:$ discarded for the classification.

**Table 3:** Accuracy of the gait pattern classification using the variance combinations of the smart shoes sensor features based on the optimal number of PCs. Pre, Acc and Gyro denote pressure sensor, accelerometer and gyroscope, respectively.

Sensor	Random Forest (%)	KNN (%)	L. Regression (%)	SVM (%)	PCs
Pressure	62.56 ±10.20	51.89 ±7.76	35.76 ±18.1	65.03 ±10.85	33
Accelerometer	53.81 ±6.95	45.21 ±5.72	50.06 ±6.33	61.19 ±8.61	33
Gyroscope	53.63 ±6.96	46.45 ±5.60	42.99 ±5.51	60.44 ±9.75	34
Pre+Acc	82.15 ±9.54	72.62 ±9.76	77.34 ±11.52	86.70 ±8.15	69
Pre+Gyro	80.54 ±10.32	74.02 ±10.54	81.29 ±10.09	86.45 ±9.41	67
**Acc+Gyro**	**85.76** ±10.02	**78.59** ±9.52	**88.69** ±6.93	**89.36** ±7.95	**68**
Pre+Acc+Gyro	84.99 ±9.72	77.52 ±10.23	87.63 ±7.52	90.64 ±6.98	100

**Table 4:** Precision of the gait pattern classification using the variance combinations of the smart shoes sensor features based on the optimal number of principal components (PCs). Pre, Acc and Gyro denote pressure sensor, accelerometer and gyroscope, respectively.

Sensor	Random Forest (%)	KNN (%)	L. Regression (%)	SVM (%)	PCs
Pressure	61.86 ±11.34	49.89 ±7.82	36.47 ±17.30	64.25 ±10.88	33
Accelerometer	53.17 ±11.73	41.43 ±6.59	47.71 ±9.45	60.36 ±9.83	33
Gyroscope	54.67 ±10.06	43.22 ±5.73	38.41 ±7.34	60.95 ±10.54	34
Pre+Acc	84.54 ±10.30	72.86 ±10.11	78.33 ±11.75	87.09 ±8.07	69
Pre+Gyro	81.71 ±10.72	74.50 ±10.45	81.49 ±11.10	86.36 ±8.44	67
**Acc+Gyro**	**87.04** ±9.94	**78.16** ±10.35	**88.30** ±7.44	**89.76** ±8.11	**68**
Pre+Acc+Gyro	86.41 ±9.78	78.23 ±10.83	87.64 ±8.36	91.08 ±6.58	100

**Table 5:** Recall of the gait pattern classification using the variance combinations of the smart shoes sensor features based on the optimal number of principal components (PCs). Pre, Acc and Gyro denote pressure sensor, accelerometer and gyroscope, respectively.

Sensor	Random Forest (%)	KNN (%)	L. Regression (%)	SVM (%)	PCs
Pressure	59.35 ±10.88	50.05 ±8.67	35.02 ±17.24	64.47 ±11.78	33
Accelerometer	44.55 ±7.69	41.50 ±6.21	41.99 ±7.24	56.73 ±9.13	33
Gyroscope	40.64 ±6.63	43.62 ±6.68	33.26 ±6.01	55.68 ±10.13	34
Pre+Acc	79.15 ±10.86	70.57 ±10.78	79.34 ±11.36	86.85 ±8.62	69
Pre+Gyro	78.23 ±11.77	70.92 ±12.11	80.92 ±10.54	86.82 ±10.41	67
Acc+Gyro	83.46 ±11.36	76.09 ±11.11	87.02 ±7.05	88.44 ±8.46	68
Pre+Acc+Gyro	83.06 ±11.18	75.12 ±11.47	88.04 ±7.59	90.55 ±7.15	100

**Table 6:** Student t-test results which compare the classification performance of SVM with random forest, KNN and logistic regression.

Methods	Accuracy	Precision	Recall
Random Forest	$\;0.008^{\;**}$	$0.24^{\;*}$	$\;0.004^{\;**}$
K-nearest neighbor	$\;0.001^{\;**}$	$\;0.001^{\;**}$	$\;0.001^{\;**}$
Logistic regression	0.397	0.291	0.520

${}^{*}\;p<0.05$; ${}^{**}\;p<0.01$.

**Table 7:** The t-test of results performance between the combination of accelerometer-gyroscope sensors against other combinations and individual types of sensors on the SVM algorithm.

Sensor	Accuracy	Precision	Recall
Pressure	$\;0.001^{\;**}$	$\;0.001^{\;**}$	$\;0.001^{\;**}$
Accelerometer	$\;0.001^{\;**}$	$\;0.001^{\;**}$	$\;0.001^{\;**}$
Gyroscope	$\;0.001^{\;**}$	$\;0.001^{\;**}$	$\;0.001^{\;**}$

${}^{*}\;p<0.05$; ${}^{**}\;p<0.01$.

**Table 8:** The effect of the number of PCs on classification performances of two and three different types of sensors.

Number of PCs	Acc+Gyro			Pre+Acc+Gyro		
	Accuracy (%)	Precision (%)	Recall (%)	Accuracy (%)	Precision (%)	Recall (%)
10	77.43	78.08	77.21	78.10	78.04	78.29
30	86.26	86.45	85.44	86.13	86.50	86.31
50	88.72	89.01	87.88	88.25	88.50	88.39
60	89.06	89.56	88.30	88.37	88.69	88.28
68 ${}^{*}$	89.36	89.76	88.44	88.51	88.84	88.39
70	89.32	89.70	88.43	89.27	89.41	89.20
90	89.30	89.78	88.34	89.29	89.78	89.29
96	89.30	89.78	88.34	89.35	89.57	89.33
100 ${}^{*}$	-	-	-	90.64	91.08	90.55
120	-	-	-	90.70	91.30	90.70
140	-	-	-	90.71	91.37	90.61
160	-	-	-	90.51	91.35	89.93
172	-	-	-	90.49	91.32	89.90

* optimal number of PC.

**Table 9:** The confusion matrix of the worst and the best performances.

Participants with the Worst Performances						Participants with the Best Performances					
**Participant 3 (average error rate of 25.4%)**						**Participant 5 (average error rate of 7.6%)**					
**Normal**	132	35	11	0	0	**Normal**	142	1	3	0	1
**Left**	37	21	0	0	2	**Left**	1	48	0	1	0
**Right**	19	0	37	0	0	**Right**	0	1	55	1	1
**Toe-out-gait**	17	0	0	38	0	**Toe-out-gait**	11	3	0	37	0
**Toe-in-gait**	0	3	0	0	50	**Toe-in-gait**	1	2	3	0	44
**Error rate (%)**	**35.6**	**64.4**	**22.9**	**0.0**	**3.8**	**Error rate (%)**	**8.4**	**12.7**	**9.8**	**2.6**	**4.3**
**Participant 8 (average error rate of 19.0%)**						**Participant 13 (average error rate of 0.9%)**					
**Normal**	123	5	2	3	10	**Normal**	140	1	0	1	0
**Left**	23	23	0	1	0	**Left**	0	54	0	0	0
**Right**	1	6	42	1	0	**Right**	0	0	52	0	0
**Toe-out-gait**	1	3	0	46	0	**Toe-out-gait**	0	0	0	52	0
**Toe-in-gait**	8	0	0	1	40	**Toe-in-gait**	1	0	0	0	56
**Error rate (%)**	**21.2**	**37.8**	**4.5**	**11.5**	**20.**	**Error rate (%)**	**0.7**	**1.8**	**0**	**1.9**	**0**
**Participant 15 (average error rate of 32.3%)**						**Participant 18 (average error rate of 0.8%)**					
**Normal**	111	9	0	9	0	**Normal**	130	0	0	0	0
**Left**	0	13	30	0	6	**Left**	1	53	0	0	0
**Right**	0	0	49	0	0	**Right**	2	1	54	0	0
**Toe-out-gait**	15	3	0	22	0	**Toe-out-gait**	0	0	0	52	0
**Toe-in-gait**	1	2	0	0	14	**Toe-in-gait**	0	0	0	0	56
**Error rate (%)**	**12.6**	**51.9**	**38.**	**29.**	**30.**	**Error rate (%)**	**2.3**	**1.9**	**0**	**0**	**0**

**Table 10:** Comparison of performance against related works.

Author	Number of Participants	Number of Classes	Method	Accuracy
Dominguez et al. [48]	6	2: supination & pronation	NN	90%
Jiang et al. [49]	8	2: jogging & walking	CNN	92.5%
Hayasi et al. [50]	13	3: healthy, L4, & L5	SVM	84.6%
Begg et al. [43]	58	2: young & elderly	SVM	90%
		2: asymptomatic,	Polynomial	67%
Mezghani et al. [51]	42	& osteoarthristis	representation	
			Wavelet	91%
Zeng et al. [52]	46	2: healthy & anterior	RBF-NN	93.47%
		cruciate ligament (ACL)		
			Counter+HMM	83.33%
Zhang et al. [53]	14	2: old & young people	Silhouette+HMM	76.24%
			Counter+Naive bayes	65.85%
			Silhouette+Naive bayes	63.28%
		Normal gait, unstable left,		
This study	18	unstable right, supination,	SVM+PCA	89.36%
		& pronation		

## Abbreviations

The following abbreviations are used in this manuscript:

Acc+Gyro	Accelerometer and Gyroscope
FN	False negative
FP	False Positive
GEI	Gait energi image
GRFs	Ground contact forces
HMM	Hidden Markov model
KNN	K-nearest neighbor
KSOM	Kohonen self-organizing mapping
LR	Logistic regression
LSS	Lumbar spinal canal stenosis
O-SVM	Optimized support vector machine
PCA	Principal component analysis
PCs	Principal components
Pre+Acc	Pressure sensor and accelerometer
Pre+Gyro	Pressure sensor and gyroscope
RBF	Radial basis function
RF	Random forest
SVM	Support vector machine
TP	True positive
TN	True negative
